# Anti-virulence strategies for *Clostridioides difficile* infection: advances and roadblocks

**DOI:** 10.1080/19490976.2020.1802865

**Published:** 2020-10-23

**Authors:** David Stewart, Farhan Anwar, Gayatri Vedantam

**Affiliations:** a Department of Surgery, University of Arizona, Tucson, AZ, USA; b School of Animal and Comparative Biomedical Sciences, University of Arizona, Tucson, AZ, USA; c Bio5 Institute for Collaborative Research, University of Arizona, Tucson, AZ, USA; d Southern Arizona VA Healthcare System, University of Arizona, Tucson, AZ, USA

**Keywords:** *Clostridioides difficile*, diarrhea, antimicrobial, virulence, antisense RNA, synthetic biologic

## Abstract

*Clostridioides difficile* infection (CDI) is a common healthcare- and antibiotic-associated diarrheal disease. If mis-diagnosed, or incompletely treated, CDI can have serious, indeed fatal, consequences. The clinical and economic burden imposed by CDI is great, and the US Centers for Disease Control and Prevention has named the causative agent, *C. difficile* (CD), as an Urgent Threat To US healthcare. CDI is also a significant problem in the agriculture industry. Currently, there are no FDA-approved preventives for this disease, and the only approved treatments for both human and veterinary CDI involve antibiotic use, which, ironically, is associated with disease relapse and the threat of burgeoning antibiotic resistance. Research efforts in multiple laboratories have demonstrated that non-toxin factors also play key roles in CDI, and that these are critical for disease. Specifically, key CD adhesins, as well as other surface-displayed factors have been shown to be major contributors to host cell attachment, and as such, represent attractive targets for anti-CD interventions. However, research on anti-virulence approaches has been more limited, primarily due to the lack of genetic tools, and an as-yet nascent (but increasingly growing) appreciation of immunological impacts on CDI. The focus of this review is the conceptualization and development of specific anti-virulence strategies to combat CDI. Multiple laboratories are focused on this effort, and the field is now at an exciting stage with numerous products in development. Herein, however, we focus only on select technologies (Figure 1) that have advanced near, or beyond, pre-clinical testing (not those that are currently in clinical trial), and discuss roadblocks associated with their development and implementation.

## Introduction

*Clostridioides difficile* infection (CDI) is a common healthcare- and antibiotic-associated diarrheal disease. If mis-diagnosed, or incompletely treated, CDI can have serious, indeed fatal, consequences. The clinical and economic burden imposed by CDI is great, and the US Centers for Disease Control and Prevention has named the causative agent, *C. difficile* (CD), as an “Urgent Threat” to US healthcare. CDI is also a significant problem in the agriculture industry. Currently, there are no FDA-approved preventives for this disease, and the only approved treatments for both human and veterinary CDI involve antibiotic use, which, ironically, is associated with disease relapse and the threat of burgeoning antibiotic resistance.^1^ Research efforts in multiple laboratories have demonstrated that non-toxin factors also play key roles in CDI, and that these are critical for disease.^2–4^ Specifically, key CD adhesins, as well as other surface-displayed factors have been shown to be major contributors to host cell attachment, and as such, represent attractive targets for anti-CD interventions.^2^ However, research on anti-virulence approaches has been more limited, primarily due to the lack of genetics tools, and an as-yet nascent (but increasingly growing) appreciation of immunological impacts on CDI.^5,6^

**Figure 1.**
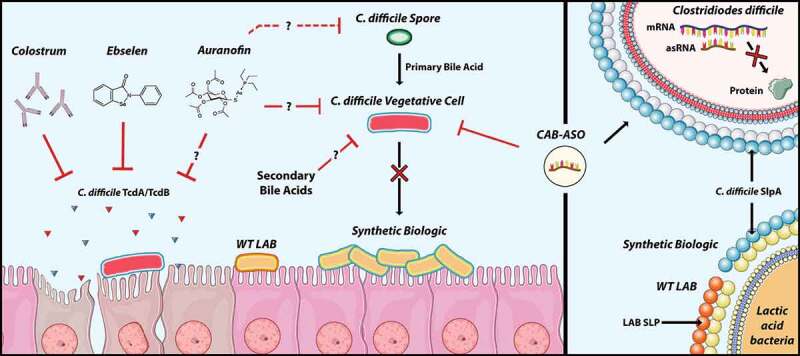
Select anti-virulence technologies to prevent or treat *Clostridioides difficile* infection. The left one-third of the schematic depicts the diseased GI tract with the small-molecule and Synthetic Biologic interventions shown. The right one-third of the figure depicts pathogen (upper) and beneficial bacterial (lower) cells and depicts targets of antisense oligonucleotide interventions (ASOs) and the Surface-Layer Protein domains used in the engineering of the lactic acid bacterial-based Synthetic Biologic. Stock images of intestinal cells (left Panel) and cell wall components (right panel) from SMART Servier Medical Art (https://smart.servier.com). CAB-ASO, cationic bolaamphiphile-antisense oligonucleotide; LAB, lactic acid bacteria; mRNA, messenger RNA; asRNA, antisense RNA, SlpA, Surface-layer Protein A, TcdA/B, *C. difficile* toxin A or Toxin B; WT, wild-type

The focus of this review is the conceptualization and development of specific anti-virulence strategies to combat CDI. Multiple laboratories are focused on this effort, and the field is now at an exciting stage with numerous products in development. Herein, however, we focus only on select technologies (Figure 1) that have advanced near, or beyond, pre-clinical testing (not those that are currently in clinical trial), and discuss roadblocks associated with their development and implementation.

## Statement of the problem

CD is an important pathogen of both veterinary and human populations.^7,8^ This gram-positive, anaerobic, spore-forming bacillus colonizes the gastrointestinal tract and can cause diarrhea; in some cases, this infection may be fatal. Risk factors in both veterinary and human populations include age, microbiota status, and prior antibiotic treatment. Disease-causing CD strains produce 1–3 toxins that enter intestinal epithelial cells and inactivate Rho family GTPases via glucosylation or ribosylation.^9^

CDI is currently the most common human healthcare-associated bacterial infection. In the USA, over 220,000 cases of CDI occur annually, costing the healthcare system over 1 USD billion.^10^ CDI is also a significant problem in the agriculture industry, with neonates (piglets, calves and foals) being particularly affected. As reviewed by Squire and Riley,^8^ and Hensgens et al.,^11^ these animals are all susceptible to CDI within 1–21 days of birth and, similar to humans, disease manifests as a severe enteritis. In swine, the greatest numbers of CD are recovered from suckling piglets in the farrowing barn.^12,13^ Further, molecular types of CD found in pigs have been recovered from human patients.^11,14^

CD epidemiology has altered markedly in the past 20 years. Virulent strains, associated with severe disease and increased recurrence rate(s), have emerged. Prevalent US human epidemic strains include North American Pulsed-Field type 1/PCR ribotype 027 (NAP1/027) and prevalent veterinary epidemic strains (now also isolated from humans) include NAP7/078.

## Challenges to conventional antibiotic therapies for *C. difficile* infection (CDI)

Conventional antimicrobial therapy targeting CD faces two distinct obstacles to the reliable eradication of this pathogen: antibiotic resistance and antibiotic-induced intestinal dysbiosis. The former is a ubiquitous challenge in the treatment of bacterial pathogens, while the latter is a challenge in the treatment of CD, with conventional antibiotics potentially leading to the population expansion of CD as well as other pathogens such as vancomycin-resistant Enterococcus (VRE). The ability of CD to sporulate only compounds these challenges. According to the US Centers for Disease Control and Prevention, approximately 2.8 million people develop an antibiotic-resistant infection annually, resulting in 35,000 annual patient deaths.^10^ Antimicrobial resistance (AMR), therefore, is a global threat, in part, due to the interconnected nature of its causal environmental, zoonotic, and anthropogenic etiologies.^15^

With respect to AMR specifically involving CD, there is evidence of clinically derived strains that are resistant to fluoroquinolones, macrolides, and cephalosporins, which may explain the frequent associations between these classes of antibiotics and CDI.^16,17^ Resistance is also observed to common CD-directed antibiotics recommended in the USA (e.g. metronidazole, vancomycin, fidaxomicin). Indeed, relatively high incidences of metronidazole resistance appear in the literature (13.3% in one study^18^) along with AMR involving vancomycin (less common) and fidaxomicin. A recent report of highly fidaxomicin-resistant CD isolates raise concern that even the more recently-introduced, narrower-spectrum antimicrobials are not being spared.^16,17^

While AMR is a frequent cause for treatment failures, CDI also has an important causal relationship with the gut environment that may be even more important in explaining the difficulties curing this infection. Specifically, antibiotics represent the single greatest risk factor for the development of CDI,^19,20^ given the ability of CD to expand its population in a dysbiotic gastrointestinal tract.^21,22^ Even the use of recommended CD-directed antibiotics may exacerbate intestinal dysbiosis,^23,24^ and multiple studies have demonstrated that commensal microorganisms may not return to “baseline” after CDI.^25,26^ These effects from conventional antibiotics require more study in human subjects to evaluate whether they contribute to persistence or recurrence of CDI, though the often-long-lasting gut ecological disturbances invoked by conventional antibiotics represent a potential, clinically relevant unintentional consequence of therapy.

## Specific challenges for anti-*C.* *difficile* interventions

Anti-virulence strategies represent a rational approach to mitigate pathogens. The idea of targeting specific CD virulence factors, rather than the whole organism, is not new. As Koo et al.^27^ and Venuto et al.^28^ have reviewed, mechanisms to precisely target those factors and/or microbes have been, and continues to be, challenging. Indeed, the very definition of “virulence” factor is now debated, since context-dependent appreciation of infectious etiology now supports the idea of a pathogenic spectrum rather than static and easily identified causative factors of disease. Additionally, any “virulence” factors, especially surface-associated molecules in bacteria, fungi or parasites may also be harbored by – or conserved in – the commensal microbiota. Therefore, amelioration of disease-causing organisms must be accomplished with extreme precision so as not to disrupt the delicate homeostasis of the normal microbiome. Specifically for CD infections, much consideration has to be given to the likelihood that humans and animals can asymptomatically harbor the organism for years.^29,30^ Any intervention may have to be administered for extended time periods to ensure complete clearance of the organism; this warrants additional considerations of safety and tolerability. Finally, many at-risk patients may be immunocompromised;^31,32^ therefore, the most appropriate interventions are those that can be tolerated by these individuals as well.

## Novel targeted strategies for CDI prevention and treatment

### *Antisense molecules with potent anti-*difficile *activity*

The two most significant disadvantages associated with the use of conventional antibiotics, AMR and a lack of organism specificity, represent two significant potential drivers for the development, persistence, and recurrence of CDI. The structure of, and mechanism of action for, conventional drugs predict these limitations thus prompting a search for alternative forms of treatment.

One such approach that has received recent attention is an effort to develop antisense antibiotics. In its simplest conceptualization, this approach involves an attempt to interdict the normal transcriptional-translational process of target bacteria, thus preventing expression of pertinent genes such as those encoding virulence factors (identified or not; Figure 1). If these genes are essential to organism survival, then their disruption will be bactericidal. Most of these approaches use an antisense oligonucleotide (ASO) designed to anneal with mRNA transcripts of the targeted gene to prevent its expression with various options available as to modify these oligonucleotides so as to become nuclease-resistant.

There are several advantages to an antisense approach, the most important of which is the potential for a treatment with *organism specificity*. Current conventional antibiotics have specific mechanisms of action, though none of these actions is specific for any particular genus or species of bacteria. This invariably results in a greater disturbance to gut bacterial communities than would be necessary to treat the pathogen of concern, leading to intestinal dysbiotic states that provide the conditions conducive to the development of CDI. Whole genome sequencing of targeted bacteria, and subsequent *in silico* analysis of its genes, allow for identification of gene pathways that may be important to bacterial survival. These data guide the construction of oligonucleotides that are of sufficient length to be both specific to a bacterial species, and that are likely to produce adequate steric inhibition in order to prevent ribosomal assembly on the target mRNA. A second potential advantage to this approach is the expansion of therapeutic targets. For example, the ability to reduce the expression of pathways responsible for bacteria-mediated intoxication has the potential to reduce the severity of CDI, thereby also reducing the need for complex medical intervention. Additionally, changing the sequence of an oligonucleotide to account for genetic variation (such as that resulting in resistance) is an inexpensive adjustment, especially by comparison to what is often required when resistance develops to conventional antibiotics.

There are no *in vivo* studies evaluating antisense antibiotics, though there are *in vitro* studies evaluating different antisense approaches. To date, the only example of antisense approaches in CD is from our (Stewart) group, where we exploited techniques used for small molecule delivery in mitochondrial medicine and applied to them to the delivery of antisense oligonucleotides.^33,34^ This technology is based upon the endosymbiotic theory of mitochondrial evolution, as well as the compositional similarities between the inner mitochondrial membrane and bacterial plasma membranes.^35,36^ With this approach, we synthesized molecules known as cationic bolaamphiphiles (CABs) that consist of two positively charged end groups separated by a hydrophobic linker of varying length; those end groups electrostatically interact with negatively-charged, and rationally designed, antisense oligonucleotides (ASOs), allowing for complexation between CAB and ASO (CAB-ASO).^37^ Electrostatic interaction between the positively-charged groups on CABs and negatively-charged phospholipids (e.g. cadiolipin) in the bacterial membrane is thought to create transient membrane pores that allow decomplexation between the CAB and ASO, introducing the antisense cargo into the bacterial cytoplasm.^38^

Multiple novel CABs have been synthesized and, by themselves, exhibit poor/negligible antibacterial activity within the concentration ranges needed to deliver their ASO cargo. However, when these CABs were combined with an ASO targeting the *dnaE* gene of CD (encoding the alpha subunit of DNA polymerase III), potent bacteridical activity was achieved at <12 µg/mL, with no detectable activity on *Escherichia coli, Enterococcus faecalis, Bacteroides fragilis*, three key representatives of the human commensal microbiota.^37^

#### Roadblocks to development

There are several challenges toward the development of this approach. Naked ASOs will not affect bacteria to a magnitude that will produce a clinical response in a human host, and thus a carrier (such as a CAB above) is required. To be effective, this carrier must perform several tasks: it must complex with the therapeutic modality to prevent both its degradation and its diffusion within the extracellular environment, delivering the ASO in a concentrated fashion to the bacterial target, while simultaneously de-complexing from the ASO at the time of delivery. The carrier must also interact with highly conserved structural elements found in bacterial plasma membranes to ensure reliable delivery of its cargo, though it should demonstrate limited-to-no antibiotic activity thus avoiding the loss of organism specificity. As opposed to eukaryotic systems, bacterial genetic variants arise much more frequently, and resistance to carriers or antisense complexes is always a possibility that needs to be formally assessed for each molecule. Additionally, evaluating other potential ASOs, and confirming that ASOs do not experience cross-inactivation from other genera of Clostridia, will require investigation.

## Synthetic biologics that curb *C. difficile* colonization and proliferation

Once CDI is established, CD toxins TcdA and TcdB are primarily responsible for the profound gut pathology and disease symptoms.^39^ Individuals with circulating antitoxin antibodies, and those who mount a rapid and effective immune response, are less prone to CDI, or experience less severe symptoms.^40,41^ With the goal of blocking toxin action to curtail intestinal damage and protect against disease; as reviewed by Riley et al.^42^ numerous groups have developed toxin-based vaccines. While such vaccine candidates may protect against disease in animal models, they typically do not decrease bacterial burdens.^42^ Bezlotoxumab, a monoclonal antibody that binds TcdB, was approved for use in patients at high risk of recurrent CDI.^43^ Other toxin-based immunization strategies are currently in various stages of clinical trials. Recently, however, Sanofi Pasteur abandoned its toxoid vaccine development since the high seroconversion (>90%) seen in Phase II failed to translate into protection against disease in a Phase III trial (https://clinicaltrials.gov).

Additionally, the remarkable efficacy of fecal transplants in treating refractory CDI, point to one unequivocal conclusion: colonization resistance is an effective and ‘natural’ method to combat CDI.^44^ Two broad therapeutic approaches that exploit colonization resistance are fecal microbiota transplantation (FMT) and probiotic administration. In randomized trials, FMT efficacy ranges from ∼50% to 90% based on delivery and number of infusions,^45–49^ but this procedure is logistically challenging and could pose undefined risks to patients;^50,51^ as such, it is recommended only for patients that repeatedly fail antibiotic therapy (at least 3 CDI episodes; IDSA-SHEA guidelines^52^). While probiotics are more palatable and pose fewer risks, they show variable efficacy in treating CDI.^53–57^ Some studies have shown probiotic efficacy when used in patients with no CDI history, but differences in formulation, dose, dosing duration and species composition preclude strong conclusions being drawn in favor of probiotics as CDI interventions. Indeed, the latest IDSA-SHEA recommendations for CDI intervention do not mention probiotics as a treatment option, and no recommendation is made for the agents in primary disease prevention.^52^

Research efforts are now being directed toward the so-called “designer” probiotics wherein specific pathogens, or pathogen niches, can be targeted to ameliorate both virulence and, in some cases, the offending organism itself. These engineered biotherapeutics show promise and many in development utilize a lactic acid bacterial platform. Lactic acid bacteria (LABs), in addition to being important members of the mammalian gut consortium, exhibit beneficial properties such as secretion of antimicrobial peptides and anti-CD activities.^56,58^ *Lactobacillus casei* can suppress the inflammatory cytokines produced in response to CDI,^59^ upregulate mucin gene expression,^60^ and also appears to confer human subjects some protection from CDI when administered as a fermented drink.^54–61^ *Lactobacillus acidophilus* has been shown to decrease CD toxin gene expression and also protect animals in a murine CDI model.^62^

Our (the Vedantam group) goal was to develop a biologic agent for colonization resistance against CDI with consistent and robust efficacy against CDI, but with a safety profile comparable to extensively used probiotics. We, therefore, sought to engineer the “Generally Regarded as Safe” (GRAS) LAB organisms to express CD surface adhesins and, thereby, competitively exclude the pathogen from intestinal surfaces.

Bacterial adherence is thought to be an important CD virulence attribute, with surface-layer proteins (SLPs) playing key roles. CD elaborates up to twenty-nine different SLPs, which are displayed in para-crystalline architecture on the cell surface,^63^ and implicated in immune modulation; thus, they are critical non-toxin virulence factors. While SLPs have been proposed as anti-CDI vaccine candidates, many groups (including ours) have reported variability in SLP epitope antigenicity;^2^ this has hampered vaccine studies. However, SlpA, a dominant CD adhesin, has a highly-conserved host-cell binding domain^3^ that we focused on for our studies (Figure 1).

LABs also harbor SLP orthologs and can express heterologous SLP molecules on their surface.^64^ We thus exploited these organisms as suitable platforms for displaying CD adhesins and engineered human probiotic LAB strains to consistently surface-display a conserved CD SlpA domain. We hypothesized that pre-colonization of the gut by engineered LAB strains in susceptible hosts would result in competitive exclusion of incoming CD. Such targeted “Synthetic Biologics” are envisaged to reduce bacterial burden and thus complement promising anti-CD toxin vaccines. As an added advantage, we postulated that extended GI tract colonization by the synthetic biologics would stimulate protective anti-CD SlpA immunity^65,66^ since neither LAB nor major gut commensals share significant SlpA sequence identity.

In our study, *L. acidophilus* and *L. casei* were genetically manipulated to express a (plasmid-encoded) chimeric SlpA consisting of a *Lactobacillus species*-derived peptidoglycan anchor, and CD-derived host-cell-binding SlpA domain (Synthetic Biologic). Golden Syrian hamster studies revealed that the engineered *L. casei* strain delayed or prevented death of animals even upon challenge with high doses of virulent CD.^67^ Colonization resistance of the Synthetic Biologic was assessed in terms of niche occupancy preventing or displacing CD establishment, and induction of an anti-CD SlpA immune response wherein serum from these animals recognized diverse CD strains. Previous studies on immunization with CD surface-layer proteins have included mixtures of multiple SLP antigens yielding mixed results in CDI prevention.^66^ Our approach is utilizing only SlpA, the most adherent CD SLP, and might therefore be more immunologically robust.

### Roadblocks to development

Lactobacilli can be extraordinarily recalcitrant to manipulation; this is both an advantage and a liability. The difficulties encountered in introducing or extracting DNA from the various species^67^ portends well for the use of the organisms in probiotic preparations due to the relatively low transformation frequency of Lactobacilli,^68^ possibly resulting in reduced risk for horizontal acquisition of antibiotic resistance genes from endogenous microbiota. Laboratory manipulation does pose unique challenges; only electroporation with high amounts (≥10 μg) of DNA, and a strain-specific optimized protocol, is the recommended method to transform *Lactobacillus* sp. Despite this, reported efficiencies may be as low as 1 transformant/μg DNA.^69^ On balance, *Lactobacillus casei* and *Lactobacillus acidophilus* offer unique advantages that can be exploited for CDI treatment. However, data regarding the strain-specific benefits of these organisms, their consistently beneficial use in diverse patient cohorts, and their ability to elicit an immune response that is protective, are scarce. Furthermore, it is unknown whether genetic manipulation of GRAS organisms will result in the re-consideration of GRAS status by regulatory pathways.

## Antibody-based therapeutics

Utilization of antibodies to treat CDI is not a novel approach; as previously mentioned, Bezlotoxumab is in use for the prevention of recurrent CDI since 2016.^70^ Colostrum is an attractive option for immunotherapy against CD due to the safety profile, relatively low cost of production and multiple forms of drug delivery,^71,72^ compared to the precise bioengineering of monoclonal antibodies. In particular, hyperimmune bovine colostrum (HBC) is of considerable interest due to the high concentrations of antigen-specific antibodies that are generated after targeted immunization of the pregnant cow. HBC has been shown to be effective in limiting symptoms and severity of several pathogen-induced GI disease including cryptosporidiosis, shigellosis, and ETEC-induced traveler’s diarrhea.^72^ Work done by Sponsellar et al.^73^ and Hutton et al.^71^ demonstrated the protective abilities of specific HBC to protect against CD infections.

Sponsellar et al. obtained HBC from a cow immunized with recombinant TcdA and TcdB.^73^ They then tested the efficacy of this HBC, in liquid or lyophilized form, to protect gnotobiotic piglets against CDI. Despite the liquid HBC preparation containing more anti-TcdA and anti-TcdB immunoglobulins, there was no overall difference between the two formulations in the protection against CD challenge. During challenge, all HBC-treated gnotobiotic piglets developed mild to resolved diarrhea, mild inflammation of the large intestine, and very little intestinal epithelial damage. In contrast, the animals treated with “non-immune” colostrum developed moderate to severe diarrhea, edema, severe neutrophilic colitis and some developed pseudomembranous colitis. Despite the clear difference in gross pathology, there was no difference in fecal or serum IL-1β and IL-8 concentrations, as well as recoverable CD between the HBC- and nonimmune colostrum-treated piglets. Promisingly, humanized gnotobiotic piglets displayed no significant difference in the diversity of the microbiome between HBC- and non-immune colostrum-treated animals.^73^

Hutton et al. generated HBC targeted toward various CD spore and vegetative cell components via immunization of pregnant cows with whole spores (Spore-HBC), exosporium (Exo-HBC), inactivated vegetative cells (Veg-HBC), Surface-Layer proteins (SLP-HBC), or the C-terminal domain of the gluocosylating toxin TcdB (TcdB-HBC).^71^ The resulting colostrum was shown to be cross-reactive to vegetative and spore surface preps, and TcdB, purified from a multitude of ribotypes. Importantly, the TcdB-HBC is able to neutralize TcdB, contributing to the increase in survival of CD infected C57BL/6 mice in a lethal infection model. This protection was seen irrespective of whether TcdB-HBC was given prophylactically or therapeutically. Interestingly, the use of any HBC treatment did not result in a decrease of recoverable CD. There was considerably less protection seen in mice given the spore/exosporium HBC or inactivated vegetative cell/SLP HBC, compared to the TcdB-HBC. However, HBC mixtures (Spore-HBC, SLP-HBC, TcdB-HBC) was demonstrated equal, if not increased, survival of CD-infected mice when compared to TcdB-HBC only treated mice. Intriguingly, this HBC-mix exhibited significant protection against recurring CDI in a mouse model of recurrence.

These data have since been corroborated through the use HBC in other animal models. Heidebrecht et al. have demonstrated that HBC, derived from cows immunized against TcdA (another CD glucosylating toxin) and TcdB, and delivered as whey proteins, provided significant protection against a CD challenge in a hamster model of acute infection.^74^ The use of immune whey has previously been shown to be effective in minimizing recurrent CDI in volunteer patients.^75,76^ Recently, Grześkowiak et al. demonstrated the use of porcine colostrum to neutralize CD TcdA and TcdB,^77^ suggesting that colostrum from multiple animal sources can be effective.

### Roadblocks to development

Colostrum has strong potential for future development as either prophylactic or therapeutic anti-virulence strategy against CDI. The low cost of production, the multiple final preparation options including those that are shelf-stable, and the ease of treatment delivery,^71^ make colostrum an attractive option for further research. However, the inability of colostrum to reliably prevent primary infection, as seen with Bezlotoxumab,^70^ means this treatment cannot be standalone. While colostrum provides an economical immunotherapy, the lack of bactericidal effect necessarily means that this strategy will have the same drawback as the monoclonal anti-toxin treatment. Mainly, there is always a risk of recurrence and spread of CD because there is no resolution of pathogen burden.

## Small molecule inhibitors of C. difficile toxins

### Ebselen

Ebselen is a synthetic organoselenium compound that possess anti-inflammatory and antioxidative properties.^78^ It has been proposed that Ebselen modifies proteins via seleno-sulfide conjugation at cysteine residues.^79,80^ In recent years, Ebselen has been reported to have antimicrobial functions against multiple clinically relevant microorganisms. Notably, Ebselen has been shown to have profound effects on multidrug-resistant staphylococcal infections; this small molecule inhibits toxin production, reduce bacterial load, and reduces established biofilms,^78^ while also acting synergistically with traditional antibiotics. Against vancomycin-resistant enterococci (VRE), Ebselen exhibited potent bactericidal activity *in vitro*, and a distinct lack of Ebselen-resistant VRE after prolonged passaging.^81^ As with multidrug-resistant staphylococcal biofilms, Ebselen was shown to significantly reduce established VRE biofilms.^81^ With the antimicrobial activity demonstrated against other notable pathogens, it becomes an attractive option to explore as an antimicrobial against CD. Work by Bender et al.^80^ and Beilhartz et al.^82^ identified and demonstrated the ability of Ebselen to inhibit the activity of CD toxins.

The large clostridial toxins of CD, TcdA and TcdB, generally share the same protein structure;^80^ there is a receptor binding domain (RBD), a translocation domain (TD), an auto-processing domain (APD), and the glucosyltransferase domain (GTD).^83^ Once the toxins bind to their cognate receptor, and are endocytosed, the acidification of the endosome results with the TD translocating the APD and GTD outside of the endosome. Inositol hexakisphosphate (IP6) binds to the exposed CPD which leads to auto-processing of the APD and release of the GTD. This GTD will then irreversibly glucosylate the Rho/Rac families of small GTPases. Ebselen has been shown to inactivate TcdA^80^ and TcdB via inhibiting the function of the APD or the GTD.^82^

Bender et al. utilized a fluorescent activity-based probe that binds to a cysteine residue of the APD active site, where upon the probe releases a stronger fluorescent signal compared to unbound probe. With this system, the group was able to indicate that Ebselen inhibited the activity of the probe by binding to the active site cysteine residue on purified APD.^80^ This result was mimicked when observing the activity of Ebselen on whole TcdA and TcdB; in a dose-dependent manner, Ebselen prevented the auto-processing and prevented the release of the GTD cleavage product. Interestingly, Ebselen was reported to not have any effect on the glucosyltransferase activity of the GTD. The inhibition of the APD was found to protect human foreskin fibroblasts from TcdB insult when the toxin was concomitantly incubated with Ebselen. Conventional Swiss-Webster mice injected with purified TcdB, pre-incubated with Ebselen (100 nM) and co-administered Ebselen, were completely protected when compared to the TcdB-only injected mice, which showed 100% lethality. Gross tissue pathology indicated less epithelial damage, neutrophilic infiltration, and submucosal edema with the addition of Ebselen treatment in a dose-dependent manner. This protection, however, did not correlate with a decrease the CD burden.^80^ Thus, Bender et al. concluded that Ebselen protects against CD via binding of the APD and preventing the release of the cytotoxic GTD.

Beilhartz et al. slightly amended these findings by indicating that Ebselen can inhibit the activity of the TcdA/TcdB glucosyltransferase domain.^82^ They engineered a recombinant TcdB with amino acid substitutions at all nine native cysteine sites (Cys-less TcdB). Despite these mutations, the recombinant TcdB was able to robustly (albeit with some delay) induce cytopathic effects on human IMR-90 (lung myofibroblasts) cells in a cytotoxicity assay. Interestingly, when wild-type TcdB and Cys-less TcdB were incubated with Ebselen, there was no difference seen in the rate of cytotoxicity.^82^ These data suggest that Ebselen-mediated protection against CD toxin insult is not solely due to the inactivation of the APD. Indeed, Ebselen was demonstrated to inhibit the glucosyltransferase activity of TcdB. More specifically, Ebselen indirectly inhibits the glucosyltransferase activity of the GTD via binding to the Rho/Rac family of small GTPases at specific cysteine residues (Cys105, Cys157, and Cys178 on Rac1) as determined by mass spectrometry. Through unknown mechanisms, the Ebselen-bound GTPase either sterically or structurally prevents the glucosyltransferase activity of the GTD.

### Repurposed gold salts

Auranofin is an orally administered gold-containing compound; it is an anti-inflammatory compound that is used for treatment of rheumatoid arthritis. Recent studies have shown potential use of Auranofin as a broad-spectrum antimicrobial.^84^ Much like Ebselen, Auranofin has been shown to be efficacious in killing or limiting virulence in numerous, clinically notable pathogens. VRE was shown to be sensitive to Auranofin-insult, resulting in a significant decrease in bacterial titers *in vitro*. Sub-inhibitory concentrations inhibit biofilm formation while mature biofilms were eradicated in a dose-dependent manner.^84^ Furthermore, Auranofin protected mice against a lethal VRE challenge and decreased the titers of recoverable VRE from these mice.^84,85^ Similar results were seen with Auranofin treatment of multidrug-resistant staphylococci.^86^

AbdelKhalek et al. recently demonstrated the antibacterial and anti-virulence effect of Auranofin on CD; specifically exhibiting bactericidal activity, inhibition of toxin production and spore formation.^87^ Use of sub-inhibitory concentrations of Auranofin resulted in a decrease in *in vitro* toxin production (40% at 0.5 MIC, determined by commercial ELISA), in a dose-dependent manner, while maintaining bacterial burden. This phenotype was also seen with sub-inhibitory concentrations of fidaxomicin, but not with vancomycin and metronidazole. Auranofin and fidaxomicin also exhibited a decrease in spore production when CD is exposed to sub-inhibitory or inhibitory concentrations. However, unlike fidaxomicin, vancomycin, and metronidazole, Auranofin was shown to protect human colorectal Caco-2 cells from CD toxin-mediated cytotoxicity.^87^ The mechanism for this protection is not known.

Hutton et al. recently corroborated these results and found Auranofin significantly reduced sporulation and toxin production *in vitro* and *in vivo*.^88^ TcdA and TcdB concentrations in culture supernatants was decreased by 5-fold in Auranofin-treated cultures compared to those that were mock-treated. In a lethal mouse model of CD infection, continuous administration of Auranofin robustly protected these mice and resulted in, at minimum, one order of magnitude reduction of recoverable spores from fecal material compared to mock-treatment. Interestingly, there was no difference in titers of recovered vegetative cells between the treatment groups suggesting that Auranofin specifically inhibits sporulation.^88^ Gross pathology of Auranofin-treated murine GI tract tissues reveals less epithelial damage (neutrophil infiltration, crypt hyperplasia, edema) and inflammation compared to mock-treated mice.

### Roadblocks to development

These data warrant further study into the mechanisms by which Ebselen and Auranofin exhibit anti-CD activity and are ideal candidates for development as novel therapies. However, studies into both compounds are extremely limited in regards to CD infections. Similar to toxin-specific immunotherapies, Ebselen and Auranofin protect against toxin insult but does not seemingly impact vegetative CD burden. Therefore, neither treatment can be standalone and another bactericidal agent is required in order to resolve infection. There are limited studies exploring the effect of Ebselen or Auranofin on CD burden *in vitro* or *in vivo*; these will need to be repeated. Furthermore, it is unknown whether other members of the gut microbiota are affected by either compound, and if such effects are detrimental to the resolution of CDI.

### Bile acid analogs

When CD spores enter a host, they survive the acid insult and are deposited within the intestinal tract. Upon recognition of certain signals by specific germinant receptors, the spore begins a germination program and subsequent outgrowth occurs.^89^ Host-derived primary bile acids, cholate (CA) and chenodeoxychole (CDCA), promote the germination of CD spores. However, microbiota-derived secondary bile acids, such as deoxycholate (DCA) and lithocholate (LCA), have been shown to inhibit germination and outgrowth.^90^ Of the organisms that can modify primary bile acids to secondary bile acids, most belong to select *Clostridium spp*.^90^ such as *Clostridium scindens*.^91^ Much like a dysbiotic microbiota, an altered bile acid profile, likely due to the loss of specific microbial communities, also contributes to the establishment of CDI.^92,93^ Restoration of the microbial-derived bile acid products, or delivery of bile acid analogs, may result in significant protection against CD.

Early work by Sorg and Sonenshein demonstrated that that the bile acid CDCA was able to diminish CD germination by acting as a competitive inhibitor to taurocholate, the primary germinant.^94^ Utilizing kinetic growth assays, they found several analogues that were able to effectively inhibit germination *in vitro*. This suggests that bile acid analogs can be utilized to minimize CD colonization and subsequent infection. Interestingly in a murine model of CD infection, murine-derived muricholic acids demonstrated inhibitory effects toward spore germination as well as vegetative cell growth.^95^ However, this potential therapy does require more interrogation; as shown by Heeg et al., CDCA does not have the same activity across multiple strains of CD,^96^ suggesting that elucidation of the inhibitory mechanism is required.

One of the secondary bile acids that can be produced by the microbiota is ursodeoxycholate (UDCA). Howerton et al. generated a cholate meta-benzene sulfonic derivative (CamSA) from UDCA that competitively inhibited taurocholate-mediated spore germination,^97^ much like CDCA. In a murine model of CD infection, CamSA was shown to prevent CDI with a single 50 mg/kg dose given concomitantly with CD spores.^98^ At lower doses, the onset of CDI was significantly delayed compared to the mock controls. Administration of CamSA severely diminished the amount of recoverable CD vegetative cells in a dose-dependent manner. Importantly, CamSA was not effective against all strains of CD, in particular the outbreak-associated strain R20291^99^ belonging to the RT027 ribotype. However, a phenyl amide analogue of CamSA was synthesized and demonstrated to be 225 times more potent at inhibiting R20291 CD spore germination compared to CDCA.^99^

#### Roadblocks to development

Bile acid analogues offer multiple strengths as therapeutics. Chief among them is that they can be synthetically refined in order to maximize their efficiency as potent inhibitors individually and synergistically, with other bile acid analogues or current traditional treatments. Despite the success of CamSA in the murine model, it was not as effective in the cricetine (hamster) model of acute CD infection, suggesting host dependent variation. While CamSA treatment delayed the onset of disease, it alone was not sufficient in preventing CDI.^100^ Of note, the microbiota was also altered after bile acid treatment. As with colostrum, Ebselen, and Auranofin, and with the view of preventing recurrent or relapsing disease, additional agents such as antibiotics may therefore be required to completely abrogate CD burden.

Taken together, more research is required to ascertain the exact mechanism behind the protective effects of each of these compounds, as well as robust elucidation of the impact of these exciting interventions on the gut microbiota.

## Concluding remarks

At the time of submission, there are currently 103 active or recruiting clinical trials involved in the treatment, prevention, or characterization of CD infection (https://clinicaltrials.gov). Of these trials, about 40% explore the effect of non-traditional therapeutic approaches such as microbial reconstitution methods (fecal microbiota transplants). Others that are of particular interest include the use of the prostaglandin analogue Misoprostol, and a Specific Carbohydrate Diet (SCD)-mediated nutritional therapy, for the reconstitution of the gut microbiota as well as the resolution of CDI. In addition, there are two CD toxin vaccines as well as multiple novel narrow-spectrum antibiotics (DNV3837, MGB-BP-3, Ridinilazole) also in, or completing, Phase II trials. These studies will help elucidate several facets of CDI such management of the disease, diminishment of bacterial burden, limiting bacterial spread, reducing emerging antibiotic resistance, and identifying key therapeutic organisms within the gut microbial community. In concert with the technologies discussed in this review, the above efforts, in sum, may provide a robust and precision-based framework to prevent and treat CD infections.
